# Supplementary material for: Effects of Fermentation with Tetragenococcus halophilus and Zygosaccharomyces rouxii on the Volatile Profiles of Soybean Protein Hydrolysates
Source: Foods. 2023 Dec 18;12(24):4513. doi: 10.3390/foods12244513 (PMC10742455; doi:10.3390/foods12244513)
Supplement: Supplementary file 1 [file foods-12-04513-s001.zip › foods-2746913-supplementary.pdf]

Table S1 Concentrations of volatile flavor compounds in different soybean protein hydrolysates analyzed by GC-MS.

Class	Number [A]	Compounds	RI	SH-0	SH-1	SH-2
Alcohols	1	Ethanol	<600	3.86±0.55[c]	328.31±4.38[a]	167.74±23.37[b]
(23)	2	2-Methyl-1-propanol	626	ND	149.05±7.78[a]	67.64±2.89[b]
	3	1-Penten-3-ol	686	0.64±0.04[a]	0.64±0.06[a]	0.60±0.03[a]
	4	Cyclopentanol	697	ND	1.84±0.03[a]	ND
	5	3-Methyl-3-buten-1-ol	729	ND	1.21±0.04[b]	2.40±0.06[a]
	6	3-Methyl-1-butanol	731	1.34±0.21[c]	854.75±22.01[b]	1026.83±145.90[a]
	7	2-Methyl-1-butanol	735	ND	135.75±2.87[b]	211.29±31.41[a]
	8	1-Pentanol	766	0.90±0.06[b]	4.17±0.17[a]	4.53±0.47[a]
	9	Prenol	775	1.57±0.01[c]	2.14±0.08[b]	4.01±0.20[a]
	10	2,3-Butanediol	792	ND	4.06±0.13[a]	0.93±0.02[b]
	11	3-Hexanol	801	ND	0.15±0.01[a]	ND
	12	4-Methyl-1-pentanol	837	ND	0.97±0.03[a]	1.16±0.01[a]
	13	1-Hexanol	869	1.35±0.19[c]	16.32±1.06[b]	19.48±2.38[a]
	14	2-Heptanol	901	ND	4.44±0.07[a]	4.18±0.02[b]
	15	1-Heptanol	971	ND	1.14±0.11[a]	0.24±0.01[b]
	16	1-Octen-3-ol	980	0.57±0.08[c]	27.52±2.26[b]	36.42±1.42[a]
	17	3-Octanol	997	9.56±1.22[a]	6.40±0.26[b]	0.50±0.05[c]
	18	2-Ethyl-1-hexanol	1030	0.81±0.11[a]	ND	ND
	19	Benzyl alcohol	1038	2.43±0.34[c]	4.69±0.13[b]	5.73±0.15[a]
	20	(E)-2-Octen-1-ol	1069	ND	4.15±0.19[a]	2.43±0.03[b]
	21	1-Octanol	1075	ND	1.36±0.01[a]	ND
	22	Linalool	1101	0.11±0.02[c]	0.29±0.01[b]	0.55±0.07[a]
	23	Phenylethyl alcohol	1117	0.86±0.02[c]	12.28±0.56[b]	32.32±3.76[a]
		**Total**		23.99±2.18[b]	1561.62±28.27[a]	1588.99±166.27[a]

Aldehydes	24	Acetaldehyde	<600	4.51±0.33[c]	7.37±0.79[a]	5.98±0.78[b]
(17)	25	2-Methyl-propanal	<600	19.16±0.93[a]	13.25±0.29[c]	16.07±0.88[b]
	26	(E)-2-Butenal	658	ND	0.43±0.05[b]	0.52±0.03[a]
	27	3-Methyl-butanal	666	277.78±21.73[a]	118.12±2.71[b]	148.41±8.84[b]
	28	2-Methyl-butanal	673	54.29±7.31[a]	34.24±1.09[b]	46.57±3.15[a]
	29	Pentanal	698	ND	ND	1.03±0.11[a]
	30	2-Methyl-2-butenal	740	1.44±0.07[a]	0.46±0.05[b]	0.38±0.04[b]
	31	3-Methyl-2-butenal	783	0.70±0.10[c]	1.27±0.05[a]	1.85±0.05[b]
	32	Hexanal	799	2.97±0.28[b]	3.90±0.03[a]	3.81±0.11[a]
	33	Heptanal	901	0.30±0.02[a]	ND	ND
	34	Benzaldehyde	963	24.19±1.93[c]	42.52±2.84[b]	62.29±6.93[a]
	35	Benzeneacetaldehyde	1048	2.41±0.23[c]	5.81±0.19[b]	8.64±0.31[a]
	36	Nonanal	1105	0.63±0.08[c]	0.77±0.02[b]	1.05±0.02[a]
	37	2,5-Dimethyl-benzaldehyde	1225	ND	5.00±0.25[a]	ND
	38	2,4-Dimethyl-benzaldehyde	1226	ND	4.27±0.15[b]	9.90±0.15[a]
	39	α-Ethylidene-benzeneacetaldehyde	1282	0.24±0.01[c]	1.23±0.02[b]	2.49±0.19[a]
	40	Dodecanal	1412	ND	ND	0.18±0.01[a]
		**Total**		388.62±16.62[a]	238.64±4.76[c]	309.18±5.27[b]
Acids	41	Formic acid	<600	0.22±0.03[b]	ND	6.26±0.29[a]
(10)	42	Acetic acid	641	0.06±0.01[c]	228.77±14.29[b]	278.65±14.88[a]
	43	Propanoic acid	702	ND	0.25±0.02[b]	0.80±0.01[a]
	44	2-Methyl-propanoic acid	760	0.32±0.03[c]	2.74±0.07[b]	7.93±0.20[a]
	45	Butanoic acid	788	0.19±0.01[c]	0.93±0.06[b]	6.96±0.14[a]
	46	3-Methyl-butanoic acid	852	2.34±0.35[b]	2.94±0.43[b]	12.46±0.60[a]

	47	2-Methyl-butanoic acid	861	ND	1.14±0.06[a]	ND
	48	Pentanoic acid	886	ND	ND	0.76±0.02[a]
	49	4-Methyl-pentanoic acid	952	ND	0.44±0.04[b]	1.30±0.09[a]
	50	Octanoic acid	1171	ND	0.84±0.10[b]	4.93±0.19[a]
		**Total**		3.13±0.32[c]	238.04±14.2[b]	320.05±15.03[a]
Esters (11)	51	Ethyl formate	<600	ND	0.22±0.03[b]	2.14±0.21[a]
	52	Methyl acetate	<600	2.34±0.04[ab]	1.90±0.18[b]	2.69±0.18[a]
	53	Ethyl Acetate	644	ND	43.16±2.67[a]	40.15±3.15[a]
	54	Propyl acetate	713	ND	0.31±0.01[a]	ND
	55	Isobutyl acetate	773	ND	0.51±0.02[b]	0.60±0.05[a]
	56	Ethyl lactate	815	ND	0.66±0.02[b]	1.78±0.12[a]
	57	Isoamyl acetate	877	ND	5.33±0.29[a]	4.28±0.27[b]
	58	2-Methylbutyl acetate	879	ND	ND	0.40±0.02[a]
	59	Benzyl acetate	1169	ND	ND	0.22±0.01[a]
	60	Phenethyl acetate	1264	ND	1.69±0.03[b]	22.15±1.19[a]
	61	γ-Nonanolactone	1372	0.11±0.02[c]	0.51±0.03[b]	1.43±0.06[a]
		**Total**		2.45±0.06[c]	54.29±2.54[b]	75.85±2.69[a]
Ketones (21)	62	Acetone	<600	266.93±26.86[a]	74.27±3.1[c]	121.17±18.13[b]
	63	2,3-Butanedione	632	1.47±0.11[b]	1.32±0.02[b]	6.92±0.39[a]
	64	2-Butanone	635	136.90±4.46[a]	61.35±2.41[c]	91.43±7.98[b]
	65	2-Pentanone	689	2.10±0.28[c]	7.63±0.44[b]	17.38±2.48[a]
	66	2,3-Pentanedione	695	0.08±0.01[c]	0.33±0.03[b]	0.59±0.01[a]
	67	3-Pentanone	679	ND	0.99±0.13[a]	ND
	68	Acetoin	710	ND	10.61±0.89[a]	ND

	69	4-Methyl-2-pentanone	736	3.68±0.46[a]	ND	ND
	70	3-Methyl-2-pentanone	749	0.24±0.02[a]	ND	ND
	71	2-Hexanone	788	0.64±0.06[a]	ND	ND
	72	2-Methylcyclopentanone	841	ND	0.24±0.01[b]	0.57±0.04[a]
	73	4-Methyl-2-hexanone	848	ND	1.12±0.03 [a]	ND
	74	2-Heptanone	891	3.44±0.51[b]	1.65±0.05[c]	26.32±0.64[a]
	75	Cyclohexanone	895	0.17±0.01[c]	0.55±0.02[a]	0.47±0.03[b]
	76	6-Methyl-5-hepten-2-one	995	0.42±0.03[b]	0.3±0.04[c]	0.56±0.01[a]
	77	Methyl cyclopentenolone	1031	ND	0.54±0.02[b]	0.61±0.02[a]
	78	Acetophenone	1071	1.36±0.06[c]	2.56±0.19[b]	3.39±0.07[a]
	79	Isophorone	1126	6.04±0.14[c]	13.35±0.66[b]	15.73±0.5[a]
	80	Benzyl methyl ketone	1132	1.12±0.13[a]	0.26±0.01[c]	0.85±0.03[b]
	81	2,6,6-Trimethyl-2-cyclohexene-1,4-dione	1148	0.31±0.00[c]	0.38±0.01[b]	0.60±0.01[a]
	82	1-(2,6,6-Trimethylcyclohexa-1,3-dien-1-yl) but-2-en-1-one	1396	0.22±0.01[c]	0.28±0.01[ab]	0.26±0.00[b]
		**Total**		431.78±24.78[a]	177.71±2.50[c]	336.11±20.28[b]
Phenols (3)	83	Phenol	984	0.37±0.05 [a]	ND	ND
	84	2-Methoxy-phenol	1093	1.28±0.03[c]	2.07±0.00[b]	2.66±0.05[a]
	85	2,4-Di-tert-butylphenol	1519	8.72±0.30[b]	9.06±0.26[b]	48.19±1.54[a]
		**Total**		10.36±0.31[b]	11.12±0.26[b]	50.85±1.50[a]
Sulfurs (6)	86	Methanethiol	<600	ND	0.46±0.04[b]	0.75±0.11[a]
	87	Dimethyl sulfide	<600	ND	0.43±0.02[a]	0.18±0.01[b]
	88	Dimethyl disulfide	742	1.37±0.06[b]	0.48±0.03[c]	3.07±0.14[a]

	89	Methional	906	1.80±0.17[c]	4.48±0.32[b]	7.27±0.76[a]
	90	Dimethyl trisulfide	974	0.35±0.02[c]	1.33±0.03[b]	2.70±0.17[a]
	91	Benzothiazole	1238	0.17±0.01[b]	0.33±0.03[a]	0.37±0.02[a]
		**Total**		3.69±0.11[c]	7.5±0.34[b]	14.33±1.01[a]
Pyrazines (19)	92	Methyl pyrazine	822	8.20±0.25[c]	9.47±0.27[a]	8.81±0.10[b]
	93	2,5-Dimethyl pyrazine	911	78.95±5.71[a]	62.35±0.67[b]	64±2.10[b]
	94	2-Ethylpyrazine	916	0.32±0.04[a]	0.18±0.01[b]	0.31±0.03[a]
	95	2,3-Dimethylpyrazine	920	0.76±0.02[a]	0.91±0.05[a]	1.04±0.06[a]
	96	2,6-Dimethylpyrazine	911	ND	0.56±0.03[b]	0.96±0.06[a]
	97	2-Ethyl-6-methylpyrazine	999	2.41±0.35[c]	5.14±0.28[b]	7.57±0.36[a]
	98	2-Ethyl-5-methylpyrazine	1002	5.42±0.06[b]	4.66±0.23[c]	5.91±0.22[a]
	99	2,3,5-Trimethylpyrazine	1004	4.04±0.53[a]	4.33±0.35[a]	4.30±0.23[a]
	100	2-Ethenyl-6-methylpyrazine	1018	1.22±0.06[b]	2.11±0.27[a]	2.07±0.12[a]
	101	2-Methyl-5-isopropyl pyrazine	1056	ND	2.72±0.07[a]	2.49±0.02[a]
	102	2-Ethyl-3,6-dimethylpyrazine	1082	4.66±0.11[c]	11.55±0.39[a]	7.52±0.03[b]
	103	2-Ethyl-3,5-dimethylpyrazine	1087	0.13±0.01[b]	0.81±0.02[a]	0.97±0.01[a]
	104	2,3-Dimethyl-5-ethylpyrazine	1090	0.54±0.04[b]	0.74±0.01[a]	ND
	105	2-Acetyl-3-methylpyrazine	1122	0.16±0.02[a]	ND	ND
	106	2,3-Diethyl-5-methylpyrazine	1159	0.14±0.01[b]	0.28±0.03[a]	ND
	107	2,3,5-Trimethyl-6-ethylpyrazine	1162	0.33±0.00[b]	0.71±0.02[a]	0.32±0.01[b]
	108	2,5-dimethyl-3-(2-methylpropyl) pyrazine	1206	0.12±0.01[a]	ND	ND
	109	2-Isoamyl-6-methylpyrazine	1257	0.11±0.01[a]	0.15±0.00[a]	ND
	110	2,5-Dimethyl-3-(3-methylbutyl) pyrazine	1321	0.26±0.02 [a]	0.26±0.01[a]	0.26±0.00[a]

		**Total**		107.77±6.69[a]	106.91±1.29[a]	106.53±1.37[a]
Furan, Pyran (8)	111	2,5-Dimethylfuran	706	ND	ND	0.16±0.01[a]
	112	2-Methyltetrahydrofuran-3-one	806	0.98±0.13[a]	0.65±0.05[c]	0.89±0.03[b]
	113	Furfural	833	0.55±0.02[c]	0.87±0.04[a]	0.73±0.03[b]
	114	2-Furanmethanol	857	14.69±2.15[b]	21.09±2.07[b]	33.43±3.72[a]
	115	5-Methyl-2-furanmethanol	956	1.15±0.07[b]	1.93±0.19[a]	2.15±0.12[a]
	116	2-Furanmethanol acetate	995	ND	ND	0.23±0.01[a]
	117	delta-Valerolactone	961	0.23±0.02[c]	0.41±0.01[b]	0.58±0.01[a]
	118	Maltol	1116	2.26±0.24[c]	4.31±0.49[b]	5.71±0.23[a]
		**Total**		19.85±2.16[c]	29.25±2.52[b]	43.88±3.94[a]
Pyridine, Pyrrole (3)	119	Pyridine	746	0.35±0.05[b]	0.72±0.02[a]	ND
	120	2,6-Lutidine	887	0.19±0.03[a]	ND	ND
	121	2-Acetylpyrrole	1065	6.29±0.56[b]	9.44±0.15[a]	10.31±0.65[a]
		**Total**		6.83±0.59[b]	10.17±0.15[a]	10.31±0.65[a]
Others (6)	122	2-Methylpentane	<600	7.62±0.48[b]	10.89±0.76[a]	4.99±0.47[c]
	123	3-Methylhexane	679	ND	0.33±0.01[a]	0.33±0.01[a]
	124	2-Methylbutyronitrile	721	0.75±0.06[a]	ND	ND
	125	3-Methylbutyronitrile	726	5.03±0.67[a]	2.02±0.07[b]	2.28±0.21[b]
	126	2,4,5-Trimethyl-1,3-oxazole	852	0.10±0.00[a]	ND	ND
	127	Benzyl nitrile	1143	0.58±0.05[b]	0.85±0.03[a]	0.97±0.03[a]
		**Total**		14.07±0.98[b]	14.09±0.85[b]	8.58±0.30[a]

Values in the same row with different letters are statistically different ($p < 0.05$) tested by one-way ANOVA and Duncan's multiple range tests.
ND, not detectable. RI represents retention index.
[A] The numbers are consistent with the compound numbers in the OPLS-DA analyses.

Table S2 The relative contents of volatile flavor compounds in different hydrolysate groups by GC-IMS.

Class	Number [A]	Compound	RI	Rt	Dt	SH-0	SH-1	SH-2
Alcohols (8)		2-Methyl-1-propanol	630	143.487	1.37527	ND	0.79±0.02[a]	0.63±0.02[b]
	128	1-Butanol	658.7	151.577	1.38604	1.39±0.03[a]	0.90±0.01[b]	0.88±0.04[b]
	129	Pentan-2-ol	704.2	166.468	1.45125	ND	0.35±0.00[a]	0.09±0.01[b]
		3-Methylbutan-1-ol	724.6	174.558	1.4874	ND	1.59±0.01[a]	1.17±0.04[b]
		2-Methylbutan-1-ol	729.9	176.749	1.47913	0.54±0.02[a]	0.46±0.00[b]	0.38±0.01[c]
	130	Pentan-1-ol-D	751.3	185.771	1.50893	0.23±0.02[c]	4.22±0.02[a]	3.43±0.09[b]
	131	Pentan-1-ol-M	757.9	188.639	1.26204	0.19±0.01[a]	0.07±0.00[b]	0.07±0.00[b]
		n-Hexanol	869.2	244.35	1.63521	0.04±0.00[a]	0.03±0.00[b]	0.03±0.00[b]
		Oct-1-en-3-ol	985.2	332.944	1.15535	1.05±0.04[b]	2.02±0.05[a]	1.98±0.01[a]
		**Total**				3.46±0.05[c]	10.42±0.07[a]	8.67±0.10[b]
Aldehydes (12)		2-Methylpropanal	548.3	122.713	1.28464	1.49±0.12[a]	0.51±0.02[b]	0.42±0.00[b]
		3-Methylbutanal	644.4	147.512	1.40763	2.78±0.00[a]	1.23±0.02[b]	1.13±0.04[b]
		2-Methylbutanal	672.8	155.74	1.40187	1.59±0.01[a]	1.00±0.01[b]	0.90±0.03[c]
	132	(E)-2-Methyl-2-butenal-M	737.2	179.753	1.09281	0.43±0.05[a]	0.05±0.00[b]	0.03±0.00[b]
	133	(E)-2-Methyl-2-butenal-D	738.1	180.144	1.3303	1.54±0.05[a]	1.34±0.01[b]	1.14±0.03[c]
		3-Methyl-2-butenal-D	776.8	197.135	1.35709	0.91±0.00[b]	1.06±0.02[a]	0.99±0.02[ab]
		3-Methyl-2-butenal-M	777.2	197.322	1.09161	0.45±0.02[a]	0.16±0.00[b]	0.13±0.01[b]
		Hexanal-D	788	202.341	1.55841	1.25±0.09[a]	0.69±0.04[b]	0.51±0.02[c]
		Hexanal-M	789.7	203.113	1.27616	1.27±0.10[a]	0.2±0.01[b]	0.17±0.01[b]
		3-Methylthiopropanal-M	904.6	266.704	1.08859	1.17±0.07[a]	0.89±0.01[b]	0.90±0.01[b]
		3-Methylthiopropanal-D	905	266.99	1.39757	0.32±0.05[c]	0.45±0.01[b]	0.60±0.02[a]
	134	(E,E)-2,4-Hexadienal-D	911.8	272.041	1.45063	0.21±0.01[a]	0.15±0.01[b]	0.12±0.01[c]
	135	(E,E)-2,4-Hexadienal-M	914.6	274.145	1.11769	1.67±0.07[a]	1.02±0.04[b]	0.87±0.05[c]

		Benzaldehyde-D	960.7	311.22	1.46809	9.06±0.07[ab]	8.62±0.16[c]	8.91±0.04[b]
		Benzaldehyde-M	962.1	312.427	1.15052	4.62±0.13[a]	2.89±0.07[b]	2.70±0.02[c]
		Benzene acetaldehyde-M	1043	404.149	1.25919	3.06±0.39[b]	3.51±0.07[a]	3.35±0.11[ab]
		Benzene acetaldehyde-D	1043	404.149	1.54054	0.30±0.04[a]	0.15±0.01[b]	0.17±0.00[b]
		Nonanal	1105	501.905	1.4862	0.66±0.08[a]	0.43±0.06[b]	0.26±0.02[c]
	136	2,4-Dimethylbenzaldehyde	1215.7	738.758	1.25992	1.67±0.11[a]	1.60±0.08[ab]	1.41±0.03[b]
		**Tota**l				34.44±0.31[a]	25.94±0.13[b]	24.71±0.26[c]
Acids (2)		Propanoic acid	721.2	173.189	1.26409	0.83±0.13[a]	0.21±0.00[b]	0.15±0.00[b]
	137	2-Methylbutanoic acid	841.2	228.953	1.21226	0.32±0.04[a]	0.09±0.01[b]	0.10±0.01[b]
		Total				1.15±0.09[a]	0.30±0.01[b]	0.25±0.00[b]
Esters (3)		Methyl acetate	547.9	122.603	1.19761	3.39±0.07[a]	2.45±0.07[b]	2.34±0.01[c]
		Ethyl Acetate	613.7	139.075	1.33773	ND	7.67±0.04[a]	6.32±0.05[b]
		Isoamyl acetate	871.7	245.72	1.7481	ND	0.52±0.02[a]	0.14±0.02[b]
		**Total**				3.39±0.07[c]	10.64±0.05[a]	8.80±0.08[b]
Ketones (10)		Acetone	502.6	112.418	1.1171	30.25±0.42[a]	22.38±0.13[b]	22.30±0.08[b]
		2-Butanone	585.3	131.722	1.24527	17.98±0.21[a]	14.12±0.05[b]	13.88±0.10[c]
		2-Pentanone	684.7	159.319	1.36763	1.08±0.03[c]	4.51±0.03[b]	5.23±0.06[a]
	138	Hydroxyacetone	708.5	168.122	1.23161	0.63±0.04[a]	0.30±0.02[c]	0.44±0.02[b]
		3-Hydroxybutan-2-one	709.4	168.494	1.32928	1.86±0.13[b]	1.28±0.04[c]	3.07±0.15[a]
		Methyl isobutyl ketone	731.6	177.438	1.17951	0.17±0.02[a]	ND	ND
	139	3-Methyl-2-pentanone	745.8	183.405	1.47687	0.22±0.02[c]	0.77±0.03[a]	0.57±0.03[b]
		2-Hexanone	778.6	197.962	1.19011	0.13±0.01[a]	ND	ND
	140	4-Heptanone	869.3	244.381	1.22715	0.58±0.05[a]	0.26±0.01[b]	0.27±0.01[b]
		2-Heptanone-D	886.6	254.397	1.62897	0.44±0.02[b]	0.22±0.02[c]	2.89±0.01[a]
		2-Heptanone-M	888.1	255.256	1.2604	0.64±0.03[a]	0.31±0.01[c]	0.46±0.01[b]
		**Total**				53.99±0.45[a]	44.13±0.10[c]	49.11±0.11[b]

Others (12)	2,6-Dimethylpyrazine	916.9	275.847	1.14188	0.17±0.01[c]	0.27±0.01[b]	0.23±0.01[a]
	Furfural	825	220.496	1.33607	0.32±0.02[b]	0.61±0.03[a]	0.56±0.02[a]
	1	849	233.103	1.62227	0.33±0.00[a]	0.15±0.00[b]	0.18±0.01[b]
	2	828.9	222.506	1.22681	1.01±0.13[a]	0.17±0.02[bc]	0.10±0.01[c]
	3	911.8	272.041	1.59065	0.16±0.00[a]	0.10±0.00[b]	0.06±0.00[c]
	4	610.9	138.34	1.29288	0.52±0.01[c]	4.81±0.03[a]	4.68±0.04[b]
	5	719.1	172.351	1.61512	0.15±0.01[c]	0.51±0.02[a]	0.34±0.02[b]
	6	904.9	266.911	1.36762	0.15±0.01[b]	0.56±0.02[a]	0.53±0.02[a]
	7	887.6	254.967	1.54856	0.20±0.02[c]	0.30±0.02[b]	0.84±0.04[a]
	8	901.9	264.699	1.66324	0.08±0.01[c]	0.22±0.02[b]	0.28±0.01[a]
	9	869.6	244.571	1.69	0.02±0.00[c]	0.25±0.01[a]	0.10±0.00[b]
	10	903.7	266.027	1.16629	0.13±0.02[c]	0.56±0.02[a]	0.53±0.01[b]
47	**Total**				3.24±0.08[c]	8.51±0.04[a]	8.42±0.05[b]

Relative content of a volatile compound was evaluated based on the percentage of each compound peak intensity out of the total peak intensity of all detected volatile compounds.

Values in the same line with different letters are statistically different ($p < 0.05$) tested by one-way ANOVA and Duncan's multiple range tests.

1 to 10 represent the unknown compound.

ND, not detectable.

[A] The numbers are consistent with the compound numbers in the OPLS-DA analyses.

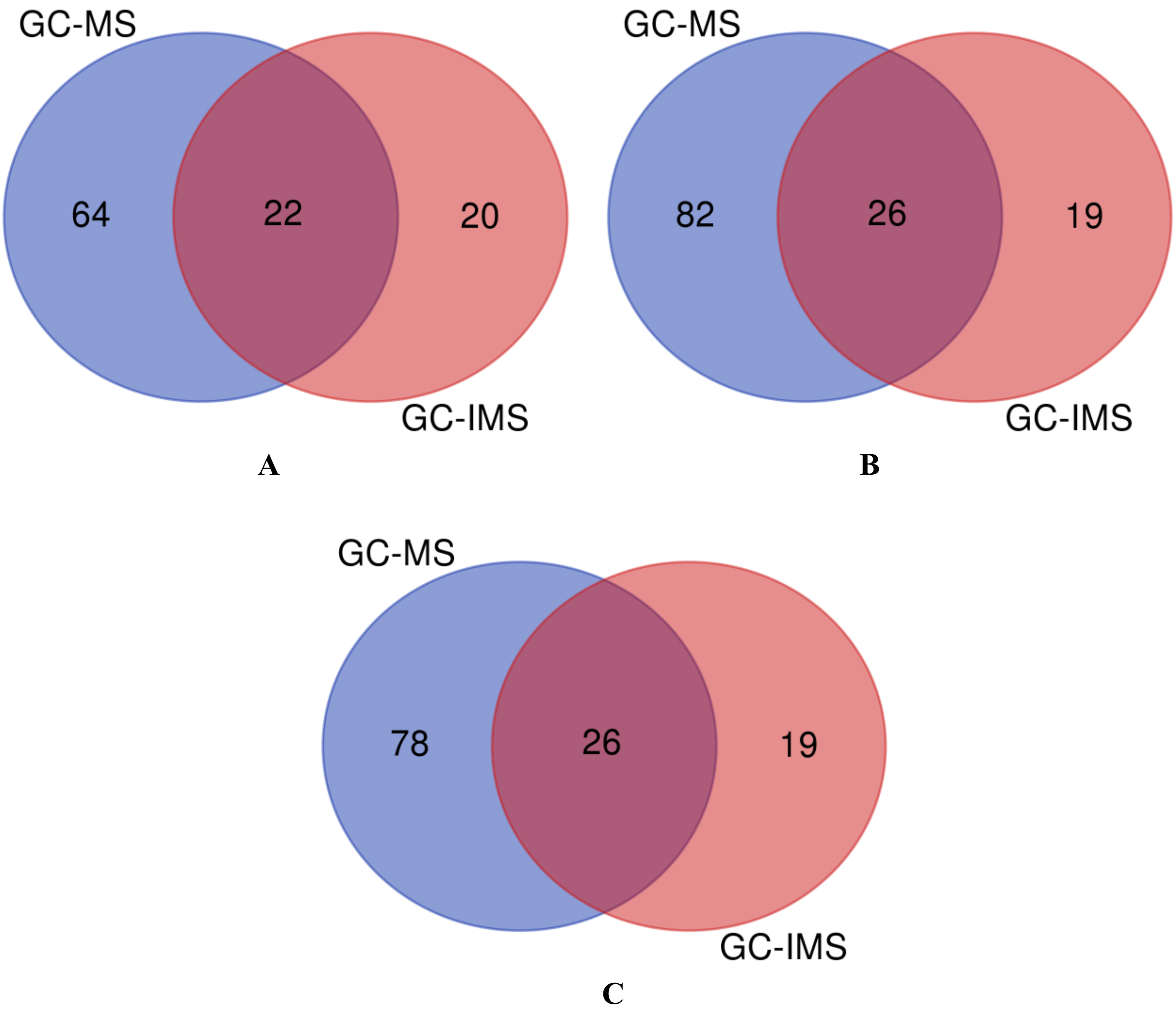

Figure S1 Venn diagrams of the volatile flavor compounds in different hydrolysate groups detected by GC-MS and GC-IMS. A, B and C represent the SH-0, SH-1 and SH-2 groups, respectively.
